# Be more, do more, research more:The importance of developing local science

**DOI:** 10.47487/apcyccv.v1i1.4

**Published:** 2020-03-30

**Authors:** Thomas F. Lüscher

**Affiliations:** 1 Royal Brompton & Harefield Hospital, Heart Division and Imperial College, National Heart and Lung Institute, London, United Kingdom London United Kingdom; University of Zurich, Center for Molecular Cardiology, Schlieren-Zurich, Switzerland. Schlieren-Zurich Switzerland

## What brought mankind forward

There were times when our ancestors lived in a dangerous environment. Indeed, primates including homo sapiens and its predecessors were neither strong nor particularly fast. They were an easy prey for predators: but they were smart and became eventually the dominant species. 

Why could this happen? First, they developed the concept of cause and effect, they learned to see events in causal terms, that one event followed another consistently and they used this to shape their environment - this made them the tool makers of the evolution. Then they discovered how to make fire and weapons and suddenly they were no longer a prey, but predators themselves. Finally, they learned to work together, to communicate, to talk to and inform each other about dangers and opportunities and to pursue them together. Working together was a key-success-factor of these social animals who alone would not have survived the struggle for survival. Thus, rational thinking, communicating with each other and working together made us the dominant species.

What brought humans forward? The ambition to be more and to do more.

## What brought Medicine forward 

Over thousand of years humans developed culture, they started to discover their body, learned how to treat wounds and injuries. Then, they tried to understand serious events such as illness and disease. They started to use herbs to treat the obvious and developed myths and beliefs for events they could not understand. Even in the 14th century when the pest hit Europe, the uncomprehensible was considered a punishment of god for the sinful. But during Renaissance a new way of understanding the unthinkable evolved: Observation and causal thinking developed what we today call science: To understand the body and its organs and with close observation as the anatomist Andreas Vesalius (1514-1564) showed with his seminal autopsies. Then William Harvey (1578-1657) concluded based on simple experiments in animals and on the veins of his forearm that the blood circulates in a pulsatle fashion in our body^1^ - the body was no longer a mystery, but an object of observation and experiments: As such modern science medicine was born.

What brought science and medicine forward? The ambition to be more and to do more.

## What has been achieved

The rise of modern medicine in the 16th century was only a start, but it changed our mindset from belief to knowledge, from assumptions to proof and from discoveries to practical applications. Of course, this process took centuries and is still ongoing, but the achievements are impressive. At first, infections were the primary target: What were the causes of the pest, cholera, smallpox and tuberculosis? In a bold experiment, Edward Jenner (1749-1823), based on the observation of many that cow pox protected from small pox, inoculated in 1757 an 8 year old boy with cow pox and the boy was subsequently immune to small pox. ^2^ Jenner called this procedure vaccination from latin word vacca, the cow. Against all odds, Robert Koch (1843-1910) and Louis Pasteur (1822-1895) convincingly showed that invisible microbes and not supranatural forces were the cause of the tuberculosis and that hygiene was the remedy. But infections remained a threat until Alexander Fleming (1881-1955) discovered Penicillin^3^ and numerous antibiotics followed, among them streptomycin. 

**Figure 1:**
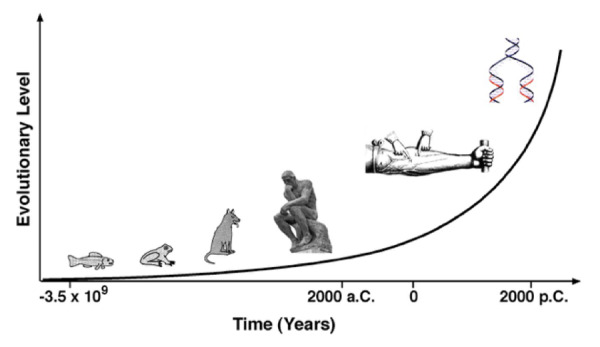
The development of scientific knowledge. Humans developed in evolution to the “thinking animal“, to discoverers and scientists up to todays molecular medicine.

After the Second World War, an English epidemiologist with the name of Austin Bradford Hill performed a seminal experiment that changed clinical research. To prove that streptomycin was indeed superior to the then established therapy with bed rest, he recruited patients with acute progressive bilateral pulmonary tuberculosis of presumably recent origin, bacteriologically proved and unsuitable for collapse therapy. He then randomized them to either treatment. As he reported in the British Medical Journal in 1948, ^(^^2^ 7% died in the streptomycin and 27% in the control group. He concluded “The difference between the two series is statistically significant; the probability of it occurring by chance is less than one in a hundred” ^(^^4^ - and as such evidence-based medicine was born.

Hill’s approach also stimulated cardiovascular medicine that became the major cause of morbidity and mortality after effective remedies against infectious disease became available. The first randomized cardiovascular trial was led by Edward Freis in patients with severe hypertension that showed in 1967 that blood pressure lowering with antihypertensive drugs reduced death, myocardial infarction and stroke. ^(^^5^ And it continued with numerous trials thereafter showing that streptokinase reduced mortality in acute myocardial infarction, ^(^^6^ that statins prevented major cardiovascular events, ^(^^7^ that anticoagulation prevented strokes in atrial fibrillation^8^ and eventually that percutaneous coronary intervention represents the treatment of choice in acute coronary syndromes. ^(^^9^ Today evidence-based recommendations are available in prevention, ^(^^10^ in intervention, ^(^^11^ for the prevention of sudden cardiac death, ^(^^12^ and valvular heart disease, ^(^^13^ in thromboembolism^14^ and heart failure^15^^)^ among other conditions - a true success story.

## What remains to be discovered

**Figure 2:**
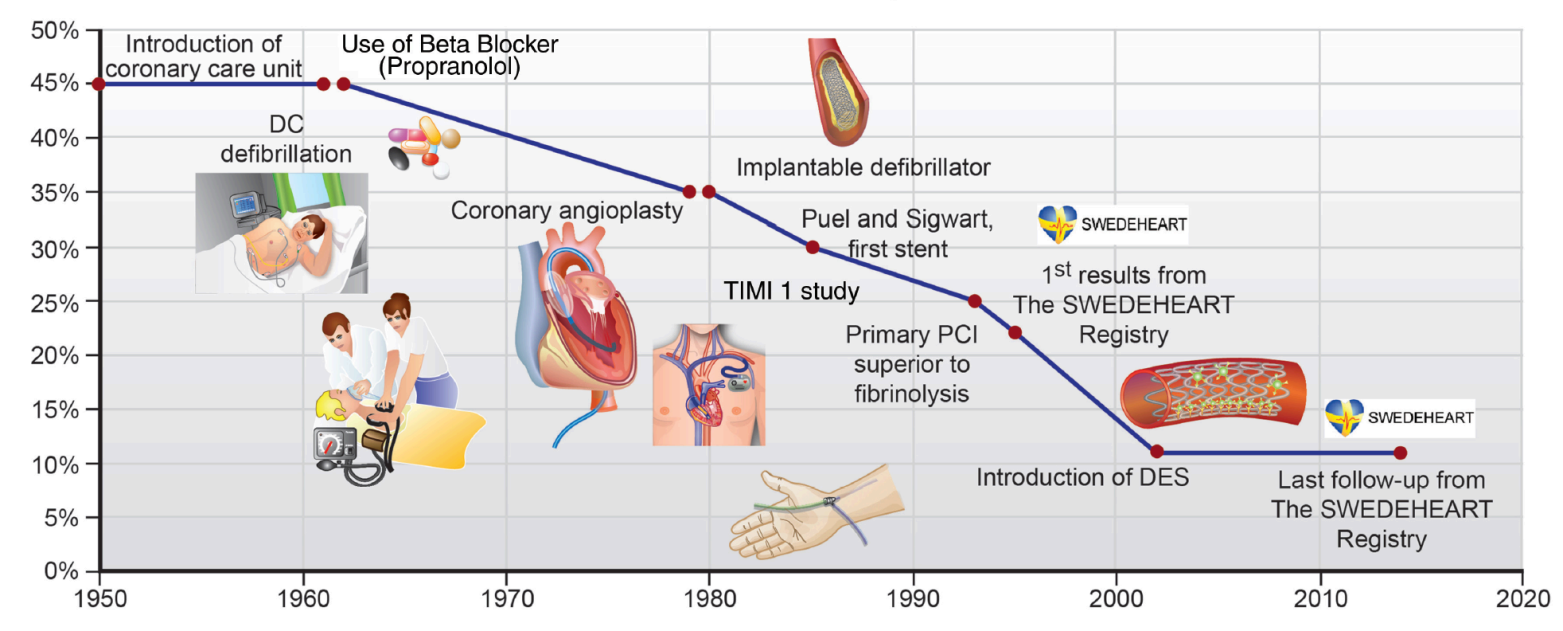
Impact of major advances in cardiovascular care on mortality in patients with acute coronary syndromes over time. From Luscher T, Obeid S. From Eisenhower's heart attack to modern management: a true success story!. Eur Heart J 2017;38(41):3066-3069.

But it does not end here: further research remains badly required, too many patients suffer from diseases that are untreatable, too many still die of conditions that are untreatable. For instance, although the history of the management of acute myocardial infarction is impressive **(**Figure 2**)**, ^(^^16^ mortality remains overall at around 10% for now due to our inability to manage cardiogenic shock appropriately. 

Also, although neurohumoral blockade and cardiac resynchronization therapy markedly reduced mortality and hospitalizations for heart failure with reduced ejection fraction or HFrEF, we are far away from a cure of the condition. Furthermore, heart failure with preserved ejection fraction or HFpEF remains an enigma without an effective treatment. ^(^^17^ Finally, although the genetics of many forms of non-ischemic cardiomyopathies are now understood, ^(^^18^ we lack effective means to correct the genetic mutation and its biological consequences in heart muscle - possibly genetic engineering will help. ^(^^19^ Another example is valvular heart disease: Yes, we can replace stenotic aortic valves surgically and now even with transarterial valve implantation or TAVI, ^(^^20^ but we have no remedy to prevent the shrinking and calcification of aortic leaflets that eventually lead to aortic stenosis. But there is more. In every country there are specific opportunities, typical unmet medical needs, unresolved scientific issues; although science is a global enterprise today, it must grow locally.

Thus, there are many unanswered questions in cardiovascular medicine that wait for young scientists and cardiologists around the world that want to be more, do more and research more.

## How to publish research

Only discoveries that are published do exist: Thus, any research needs to be finished, written up and submitted to a scientific journal. Only what can be read by others, will advance science and medicine. ^21^

Most journals, and in particular the best and most respected, work based on the peer review system, i.e. they ask expert in the field to evaluate the submitted work to make suggestions, to provide constructive cirticism, in an attempt to make good papers even better and to reject those who do not make the bar. What are the criteria editors use when assessing submitted work? **(**Table 1**)** First, innovation: Research is about new findings, about expanding our knowledge base and about new treatment targets and novel remedies for disease. Second, precision: Research must be precise, performed with state-of-the-art equipment. Third, stringency: Research must provide proof, supply data that convince the sceptics and are accepted by experts. Forth, honesty: Research builds on trust of others, only correctly obtained data are reproducible; and the scientific process is build on reproducibility of findings. Fifth and last, be in time: The scientific process is competitive, you are not the only one working in your field of interest; indeed, most scientists who ever worked in history work today. 

**Table 1:** Major Criteria of Good Science

Criterion	Remarks
Innovation	Research is about novelty either as a true discovery or as incremental innovation or more solid evidence with a larger cohort, longer follow-up or better mechanistic insight.
Precision	Measurements should be made with state-of-the-art equipment. If possible, mechanistic insight and causality should be provided.
Stringency	Data presentation should follow a logical stream in order and regards explanation of the findings.
Honesty	Science relies on proper and correct data reporting; anything else is not scientifically correct research.
Timeliness	Science is a global endeavor and hence there is a lot of competition. Being in time, means being first.
Reference	Give credit in you manuscript to those who work in your field, cite those who set the basis of your work - they may be your reviewers.

## How to become successful

If you want want to be more, do more and research more, what do you have to do? First, one needs proper training in an excellent institution with mentors devoted to education of the next generation - the better the mentor, the more you will grow. Second, you must find out what fascinates you the most as you are only good at things you like. Third, you must read the current literature as you can only provide innovation if you know what is already known. Forth, forget life-work balance in the first years of your career; you only can take off, if you invest enough energy, creativity and time. Fifth, visit and take part in congresses and courses of the highest quality to learn from and meet the best in your field. Networking is important. Finally, plan a stay abroad in an instiution of excellence in your field of interest - it will boost your professional and personal development.
